# Supplementary material for: Using deep clustering to improve fMRI dynamic functional connectivity analysis
Source: Neuroimage. 2022 Aug 15;257:119288. doi: 10.1016/j.neuroimage.2022.119288 (PMC10751537; doi:10.1016/j.neuroimage.2022.119288)
Supplement: Supplementary Data S1 — Supplementary Raw Research Data. This is open data under the CC BY license http://creativecommons.org/licenses/by/4.0/ [file mmc1.pdf]

Supplementary Materials

Generating State FC Matrices and Transition Matrices

To generate each FC matrix, we chose the number of modules it should comprise a priori. We set the maximum module number to 5, 7 and 9 when generating data with 15, 25 and 50 nodes respectively (increased from 3 modules used when generating data with 10 nodes in the original code (Allen et al., 2014)). Then, each node was randomly assigned a sign (+/-) and a module number, with uniform probability. The FC matrix was then constructed by assigning a positive connection where nodes belonged to the same module and had the same sign, and a negative connection between nodes which belonged to the same module and had different sign. We then re-ordered the nodes such that the matrix had optimal community structure, using the *modularity_und* function from the Brain Connectivity Toolbox (`https://sites.google.com/site/bctnet/`) (Rubinov and Sporns, 2010), in order to produce dFC states with a visible modular structure to allow qualitative comparison of state FC matrices. This process was repeated for each state.

To generate the transition matrix governing the HMM, the probability of remaining in the same state s was initially set to a value p_s (i.e. values on the leading diagonal of the transition matrix). The remaining $1 - p_s$ was then randomly split between all states (including state s in order to induce different dwell times in different states). The value of p_s was determined by experimenting with values close to 1, in order to give a median switching timescale of around 60 s, and was varied for datasets with different number of states. We set $p_s = 1 - 0.005 \times n$, where n is the number of states. Supplementary Fig. 1 demonstrates the variability in switching timescales in a synthetic dataset.

References

Elena A Allen, Eswar Damaraju, Sergey M Plis, Erik B Erhardt, Tom Eichele, and Vince D Calhoun. Tracking whole-brain connectivity dynamics in the resting state. *Cerebral cortex*, 24(3): 663–676, 2014.

Erik B Erhardt, Elena A Allen, Yonghua Wei, Tom Eichele, and Vince D Calhoun. Simtb, a simulation toolbox for fmri data under a model of spatiotemporal separability. *Neuroimage*, 59 (4):4160–4167, 2012.

Karl J Friston, Andrew P Holmes, Keith J Worsley, J-P Poline, Chris D Frith, and Richard SJ Frackowiak. Statistical parametric maps in functional imaging: a general linear approach. *Human brain mapping*, 2(4):189–210, 1994.

Karl J Friston, Andrea Mechelli, Robert Turner, and Cathy J Price. Nonlinear responses in fmri: the balloon model, volterra kernels, and other hemodynamics. *NeuroImage*, 12(4):466–477, 2000.

Mikail Rubinov and Olaf Sporns. Complex network measures of brain connectivity: uses and interpretations. *Neuroimage*, 52(3):1059–1069, 2010.

Noise level	Unique event probability	Unique event amplitude	Gaussian noise amplitude
Low	$p_u \sim \mathcal{N}(0.3, 0.2^2)$	$a_u \sim \mathcal{N}(0.3, 0.2^2)$	$a_g \sim \mathcal{N}(0.3, 0.2^2)$
Medium	$p_u \sim \mathcal{N}(0.5, 0.3^2)$	$a_u \sim \mathcal{N}(0.5, 0.3^2)$	$a_g \sim \mathcal{N}(0.4, 0.2^2)$
High	$p_u \sim \mathcal{N}(0.6, 0.2^2)$	$a_u \sim \mathcal{N}(0.7, 0.2^2)$	$a_g \sim \mathcal{N}(0.6, 0.2^2)$

Supplementary Table 1: Parameters used to generate synthetic data with low, medium and high noise levels. In order to create heterogeneous subjects, each parameter was sampled from a normal distribution for each subject. The distributions used are shown for the unique event probability, p_u, unique event amplitude, a_u, and Gaussian noise amplitude, a_g, each displayed in the form $\mathcal{N}(\mu, \sigma^2)$ with a mean μ and standard deviation σ. Examples of node timeseries with each noise level are shown in Supplementary Fig. 3. See the main text for explanation of these parameters in the SimTB model.

HRF Model	Parameter	Value/Distribution
Canonical	Delay of response	$4.0 + \lvert\mathcal{N}(0, 3.0^2)\rvert$
	Delay of undershoot	$12.0 + \lvert\mathcal{N}(0, 3.0^2)\rvert$
	Dispersion of response	$\mathcal{N}(1.0, 0.2^2)$
	Dispersion of undershoot	$\mathcal{N}(1.0, 0.2^2)$
	Ratio of response to undershoot	$2.0 + \lvert\mathcal{N}(0, 3.0^2)\rvert$
	Onset (s)	0
	Length of kernel (s)	32
Winkessel-Balloon	Signal decay	$1/\mathcal{N}(1.54, 0.15^2)$
	Autoregulation	$1/\mathcal{N}(2.46, 0.15^2)$
	Transit time	$\mathcal{N}(0.98, 0.15^2)$
	Stiffness	0.36
	Oxygen Extraction Fraction	0.34
	Neural efficacy	0.54

Supplementary Table 2: Values and distributions of the parameters of each HRF model implemented in SimTB. These are the defaults used in the SimTB code; detailed explanation of each parameter is found in Friston et al. (1994) for the canonical HRF, or Friston et al. (2000) for the Windkessel-Balloon HRF. Parameters with distributions were randomly sampled for each subject.

Nodes	Dimensionality	p
15	105	[**16**, 32, 64]
25	300	[16, **32**, 64, 128]
50	1225	[16, 32, 64, 128, **256**, 512]

Supplementary Table 3: Parameter values searched when tuning the number of principle components, p, to be used for clustering following dimensionality reduction with PCA. For each number of nodes in the parcellation, the dimensionality of the dFC matrices is shown, as well as the range of parameters searched during tuning. The values which gave maximal clustering accuracy in synthetic training data are shown in bold for each number of nodes.

Nodes	Dimensionality	m	v	u
15	105	[**30**, 40, 50, 60]	[0.1, 0.2, 0.5, **1.0**]	[**32**, 64]
25	300	[**30**, 40, 50, 60]	[0.1, 0.2, 0.5, **1.0**]	[32, **64**]
50	1225	[30, **40**, 50, 60]	[0.1, 0.2, 0.5, **1.0**]	[**32**, 64, 128]

Supplementary Table 4: Parameter values searched when tuning the UMAP algorithm for dimensionality reduction. A grid search was performed over all combinations of values for these three parameters. m is the number of neighbours used to determine the local connectivity of the high-dimensional graph before optimising the low-dimensional representation, v is the minimum permissible distance between points in the low-dimensional representation, and u is the number of dimensions. See the main text for explanation of these parameters. For each number of nodes in the parcellation, the dimensionality of the dFC matrices is shown, as well as the range of parameters searched during tuning. The values which gave maximal clustering accuracy in synthetic training data are shown in bold for each number of nodes.

Nodes	Dimensionality	d_1	d_2	d_3
15	105	[128, 256, **512**]	[64, 128, **256**]	[**16**, 32, 64]
25	300	[128, 256, **512**]	[64, 128, **256**]	[16, **32**, 64]
50	1225	[256, 512, **1024**]	[128, **256**, 512]	[16, 32, **64**]

Supplementary Table 5: Parameter values searched when tuning the autoencoder architecture for deep clustering. A grid search was performed over all combinations of values for these three parameters. d_1, d_2 and d_3 are the number of units in the layers of the symmetric autoencoder (giving d_3-dimensional encoded data). For each number of nodes in the parcellation, the dimensionality of the dFC matrices is shown, as well as the range of parameters searched during tuning. The values which gave maximal clustering accuracy in synthetic training data are shown in bold for each number of nodes.

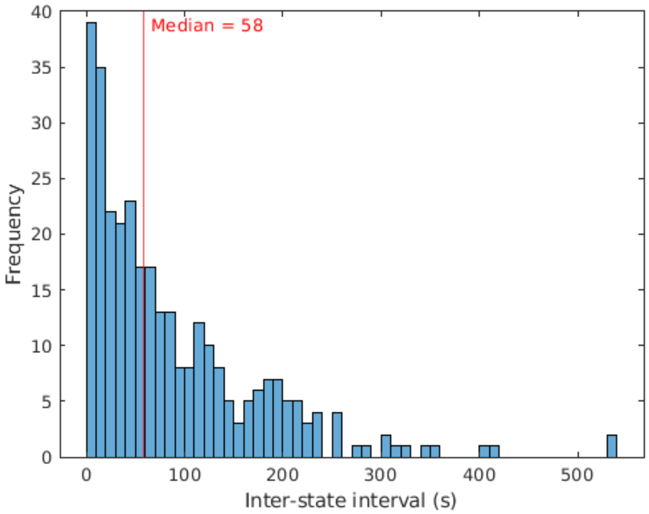

Supplementary Figure 1: Demonstration of the variability in switching timescales in a synthetic dataset. The histogram shows the frequency of inter-state intervals (the time between state changes) in one synthetic dataset containing 50 subjects, each with a timeseries duration of 540 s.

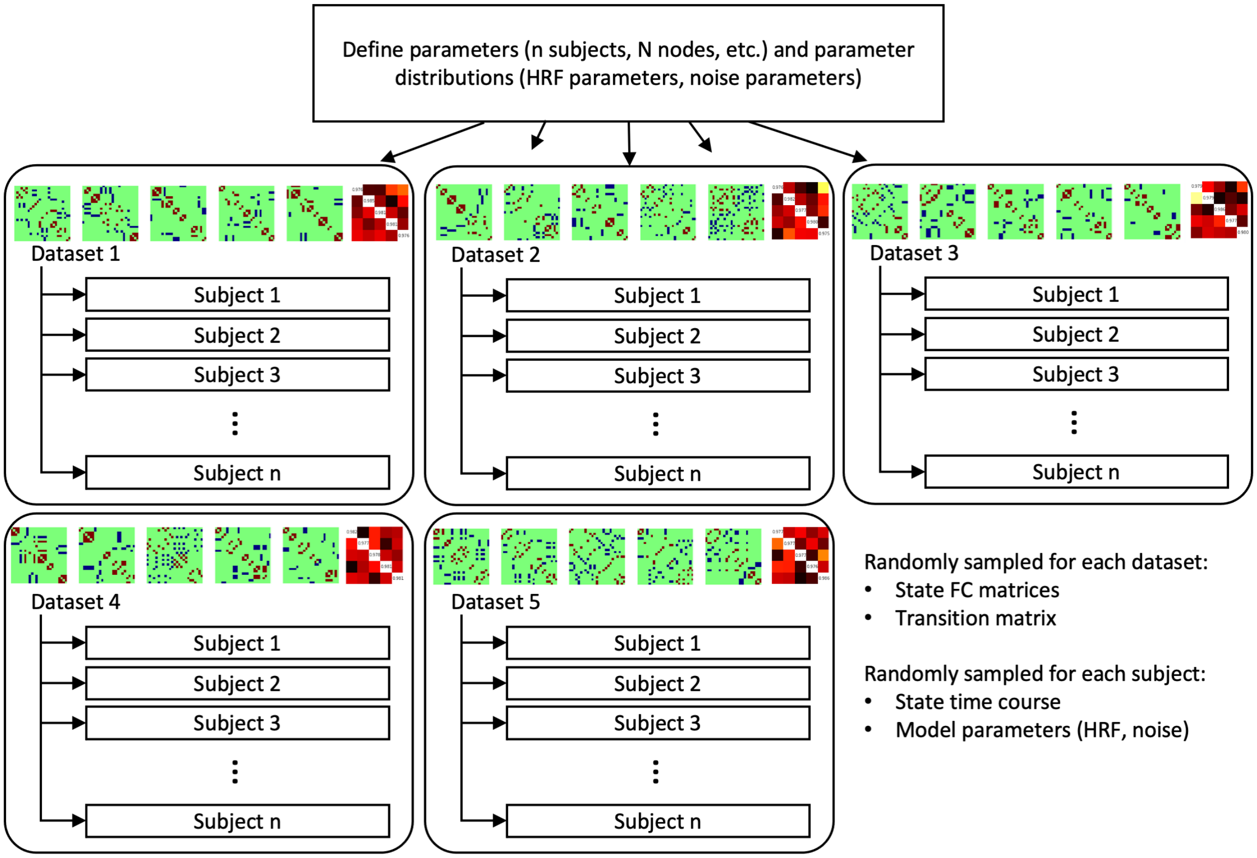

Supplementary Figure 2: Model hierarchy for generating synthetic datasets. First the parameter set is defined (including the number of subjects, number of nodes and distributions for noise and HRF parameters), then datasets can be generated, each with a unique set of state FC matrices and a unique transition matrix. Each dataset contains multiple subjects, which each have a randomised time course of underlying states, sampled from a HMM governed by the transition matrix, and HRF and noise parameters sampled from the predefined normal distributions. HMM = hidden Markov Model; HRF = hemodynamic response function.

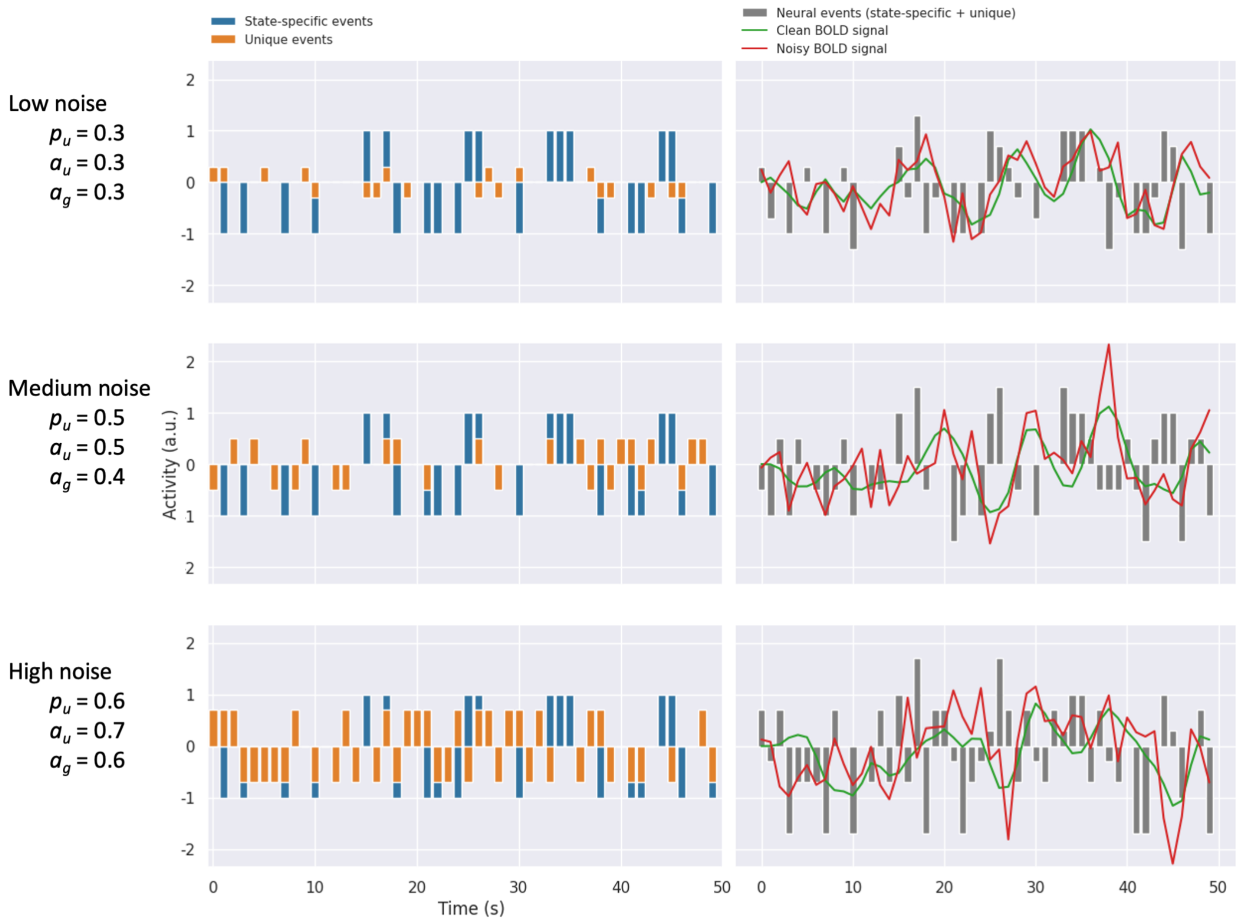

Supplementary Figure 3: Demonstration of the effect of different noise parameters in the SimTB model. Each plot show simulated activity from a single node in a single subject, with low noise (top), medium noise (middle), or high noise (bottom). Parameter values for the unique event probability (p_u) and amplitude (a_u) and the Gaussian noise amplitude (a_g) are shown. For this figure these parameters were set to the mean of the distribution shown in Supplementary Table 1. The time course of state-specific events (the neural events which drive the functional connectivity) is the same in all these plots. Unique events (which represent spurious, node-specific neural events) were sampled according to the parameters shown. Neural events were summed at each time point (state-specific + unique), then convolved with the hemodynamic response function, giving the "clean" BOLD signal. Gaussian noise was then added to give the final, noisy BOLD signal.

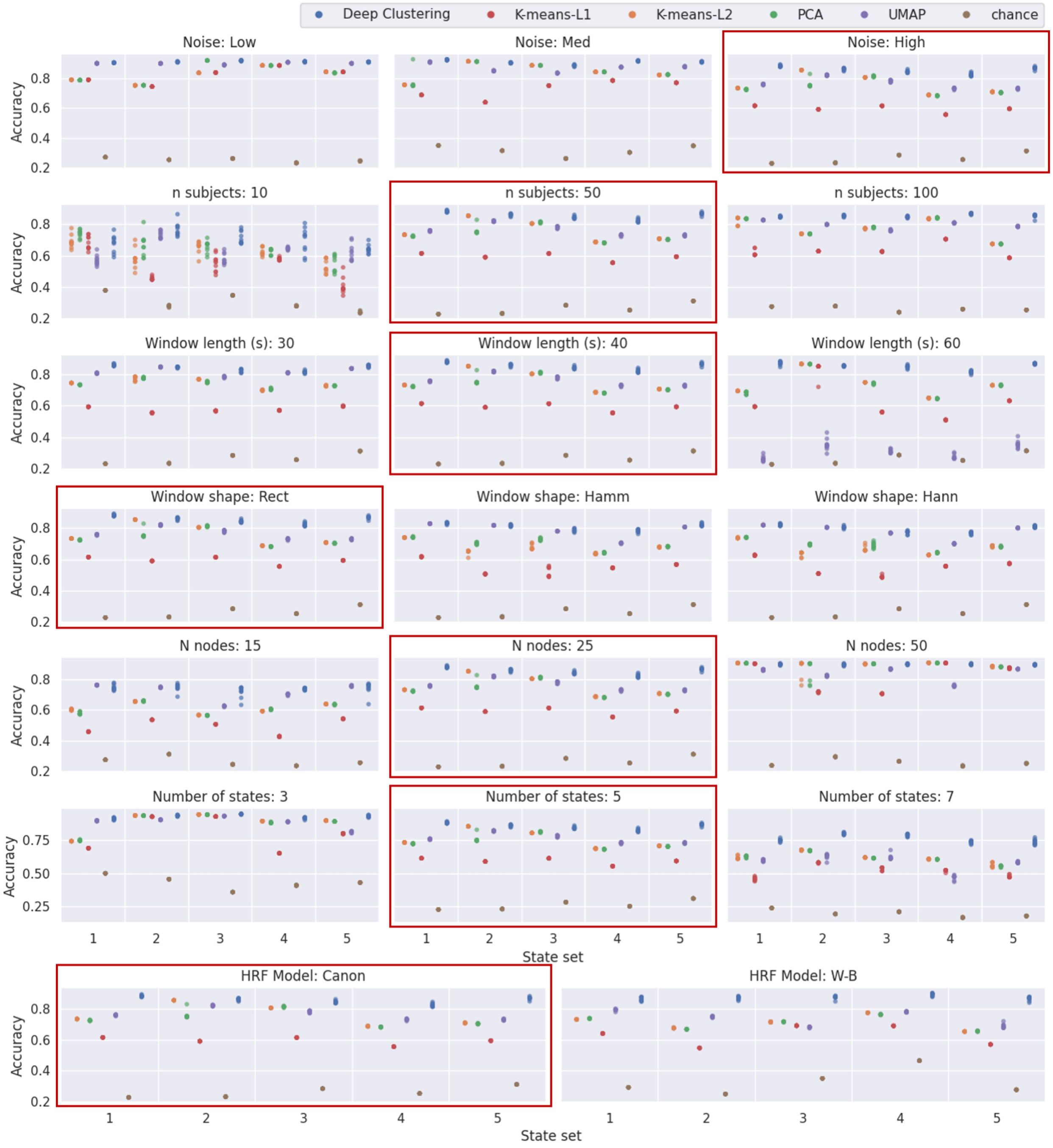

Supplementary Figure 4: Clustering accuracy in synthetic data. For each parameter set, five unique sets of states were randomly generated, each with a corresponding randomly generated transition matrix. Each method was applied to each dataset 10 times. The accuracy is plotted for each run of each method on each set of states with each parameter set. The red boxes indicate the default parameter sets, thus each of these plots show the same data, but are replicated to allow comparison with adjacent plots in which parameters are varied. Rect = rectangular; Hamm = Hamming; Hann = Hanning; HRF = hemodynamic response function; Canon = canonical; W-B = Windkessel-Balloon.

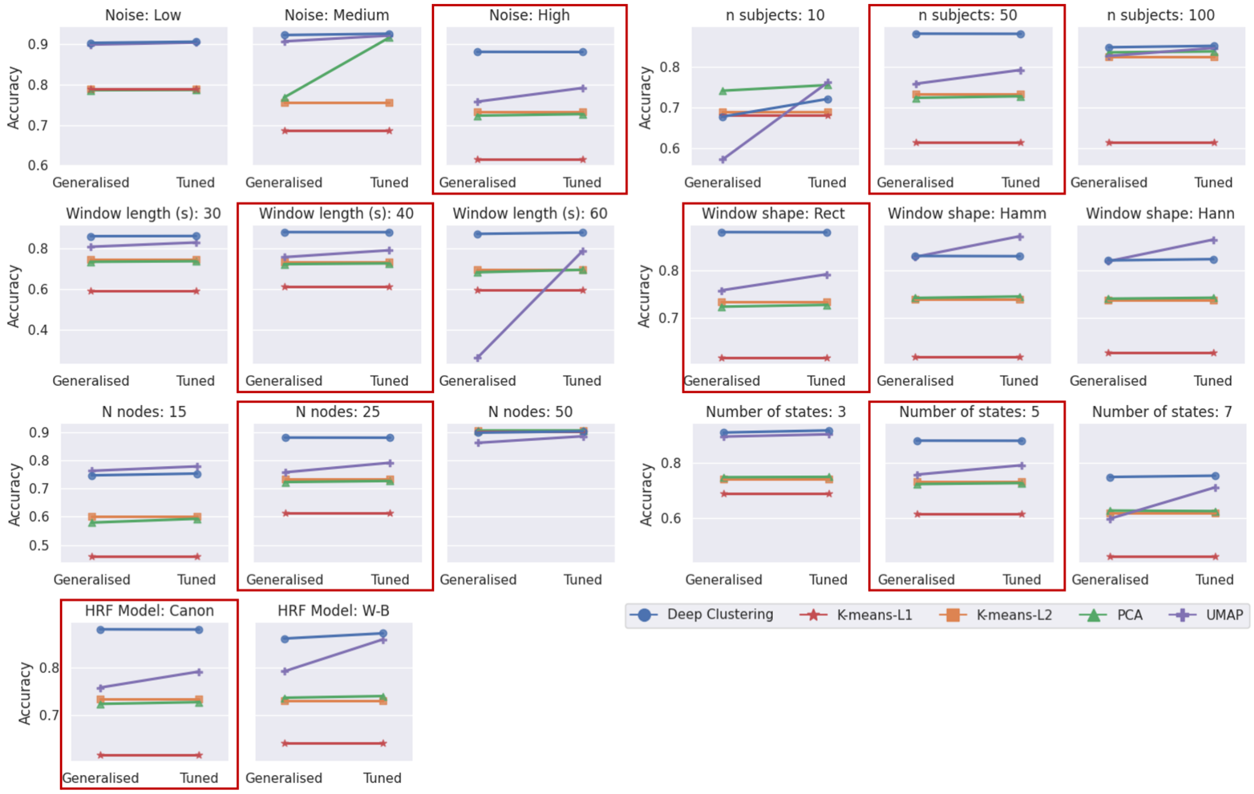

Supplementary Figure 5: Comparison between clustering accuracy with generalised hyperparameters, and clustering accuracy with hyperparameters tuned to the validation data. Generalised accuracy was measured using hyperparameters given in Table 1 in the main text, which were tuned using a separate tuning datatset. Tuned accuracy was measured using hyperparameters tuned on the validation data, indicating the upper bound of the accuracy for each method. Raw k-means with L1 or L2 distance requires no parameter tuning, therefore generalised and tuned accuracies are the same for these methods. The red boxes indicate the default parameter sets, thus each of these plots show the same data, but are replicated to allow comparison with adjacent plots in which parameters are varied. Rect = rectangular; Hamm = Hamming; Hann = Hanning; HRF = hemodynamic response function; Canon = canonical; W-B = Windkessel-Balloon.

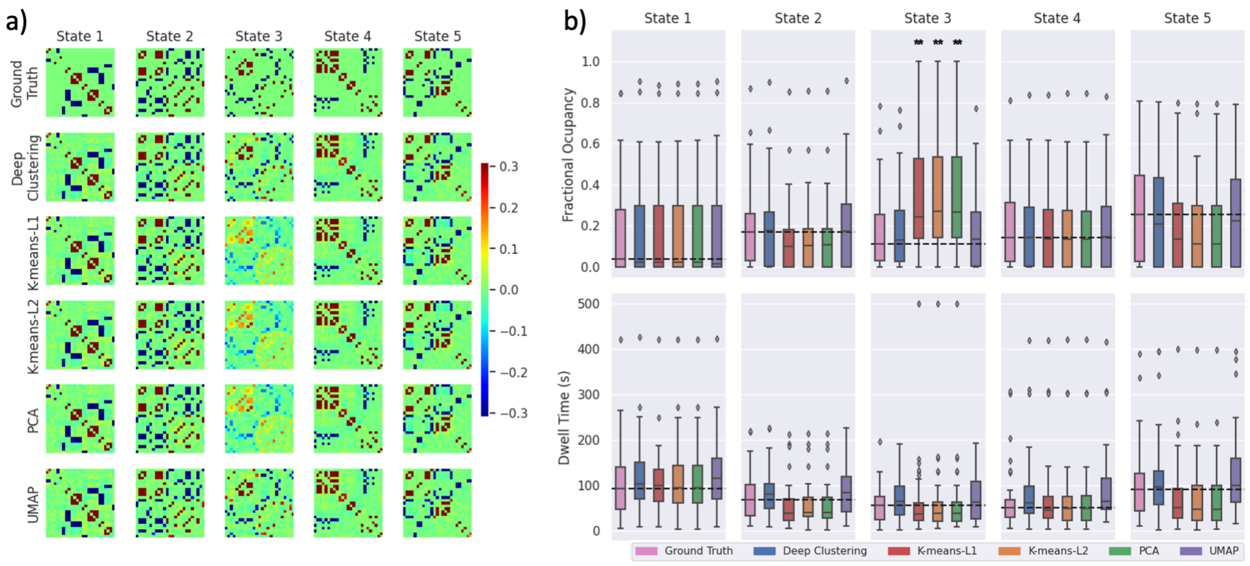

Supplementary Figure 6: Clustering results from one run of each dimensionality reduction method applied to synthetic data with low noise. a) State FC matrices are plotted with connectivity indicated by the colour bar. b) The distribution across subjects of fractional occupancy and dwell time measurements are plotted for each state. Boxes show the interquartile range, with a line for the median. The median of the ground truth is shown as a dashed line across each plot for comparison with other methods. Whiskers extend to the range of the data, not including outliers which are shown as diamonds. Significant differences from the true distributions, measured by unpaired two-tailed t-tests, are indicated as follows: $*p < 0.01$, $**p < 0.001$, $***p < 0.0001$.

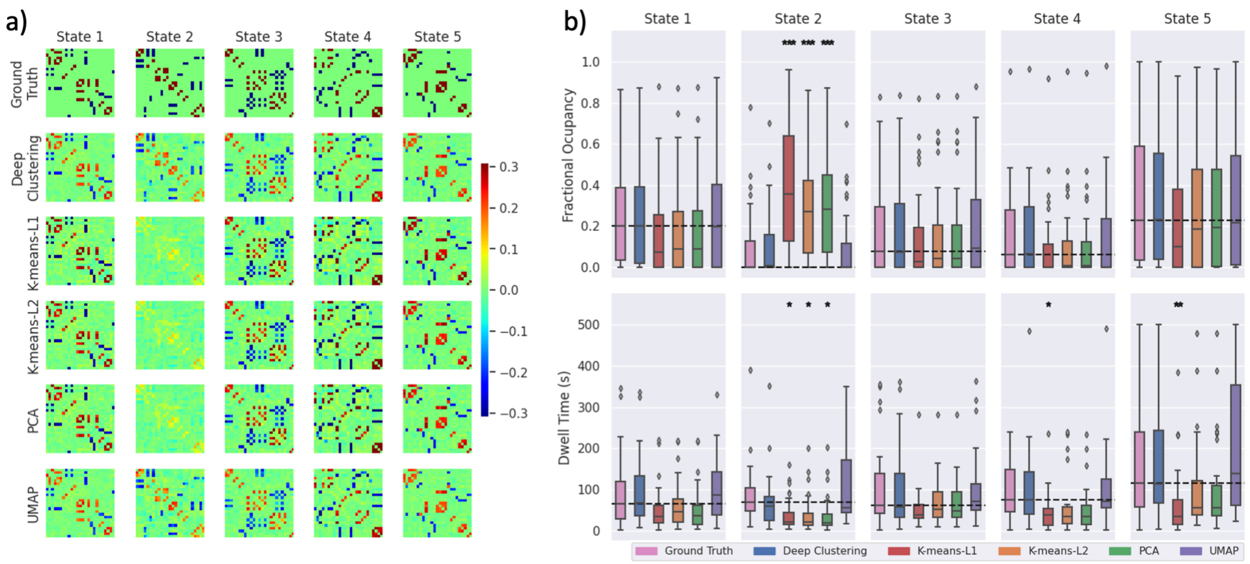

Supplementary Figure 7: Clustering results from one run of each dimensionality reduction method applied to synthetic data with medium noise. a) State FC matrices are plotted with connectivity indicated by the colour bar. b) The distribution across subjects of fractional occupancy and dwell time measurements are plotted for each state. Boxes show the interquartile range, with a line for the median. The median of the ground truth is shown as a dashed line across each plot for comparison with other methods. Whiskers extend to the range of the data, not including outliers which are shown as diamonds. Significant differences from the true distributions, measured by unpaired two-tailed t-tests, are indicated as follows: $*p < 0.01$, $**p < 0.001$, $***p < 0.0001$.

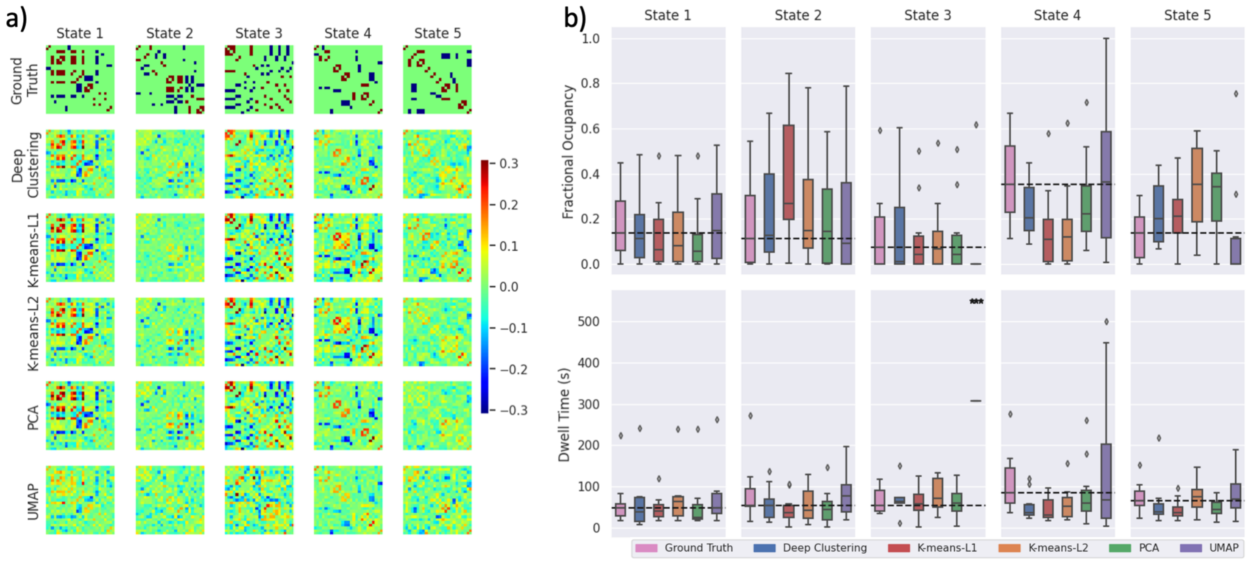

Supplementary Figure 8: Clustering results from one run of each dimensionality reduction method applied to synthetic data with 10 subjects. a) State FC matrices are plotted with connectivity indicated by the colour bar. b) The distribution across subjects of fractional occupancy and dwell time measurements are plotted for each state. Boxes show the interquartile range, with a line for the median. The median of the ground truth is shown as a dashed line across each plot for comparison with other methods. Whiskers extend to the range of the data, not including outliers which are shown as diamonds. Significant differences from the true distributions, measured by unpaired two-tailed t-tests, are indicated as follows: $*p < 0.01$, $**p < 0.001$, $***p < 0.0001$.

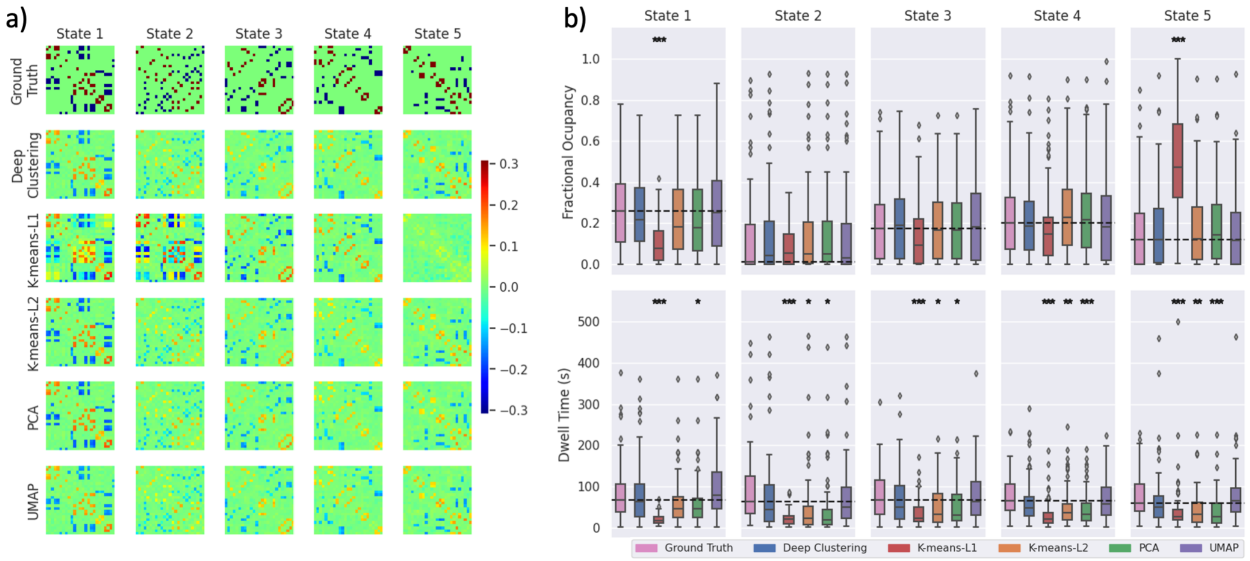

Supplementary Figure 9: Clustering results from one run of each dimensionality reduction method applied to synthetic data with 100 subjects. a) State FC matrices are plotted with connectivity indicated by the colour bar. b) The distribution across subjects of fractional occupancy and dwell time measurements are plotted for each state. Boxes show the interquartile range, with a line for the median. The median of the ground truth is shown as a dashed line across each plot for comparison with other methods. Whiskers extend to the range of the data, not including outliers which are shown as diamonds. Significant differences from the true distributions, measured by unpaired two-tailed t-tests, are indicated as follows: $*p < 0.01$, $**p < 0.001$, $***p < 0.0001$.

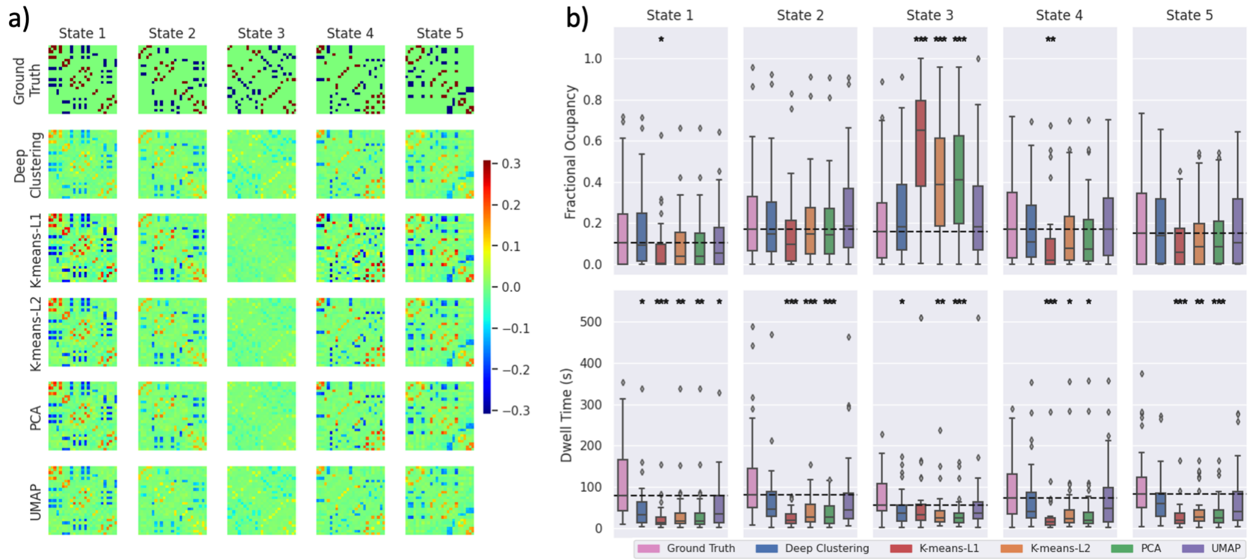

Supplementary Figure 10: Clustering results from one run of each dimensionality reduction method applied to synthetic data processed with a 30 s window. a) State FC matrices are plotted with connectivity indicated by the colour bar. b) The distribution across subjects of fractional occupancy and dwell time measurements are plotted for each state. Boxes show the interquartile range, with a line for the median. The median of the ground truth is shown as a dashed line across each plot for comparison with other methods. Whiskers extend to the range of the data, not including outliers which are shown as diamonds. Significant differences from the true distributions, measured by unpaired two-tailed t-tests, are indicated as follows: $*p < 0.01$, $**p < 0.001$, $***p < 0.0001$.

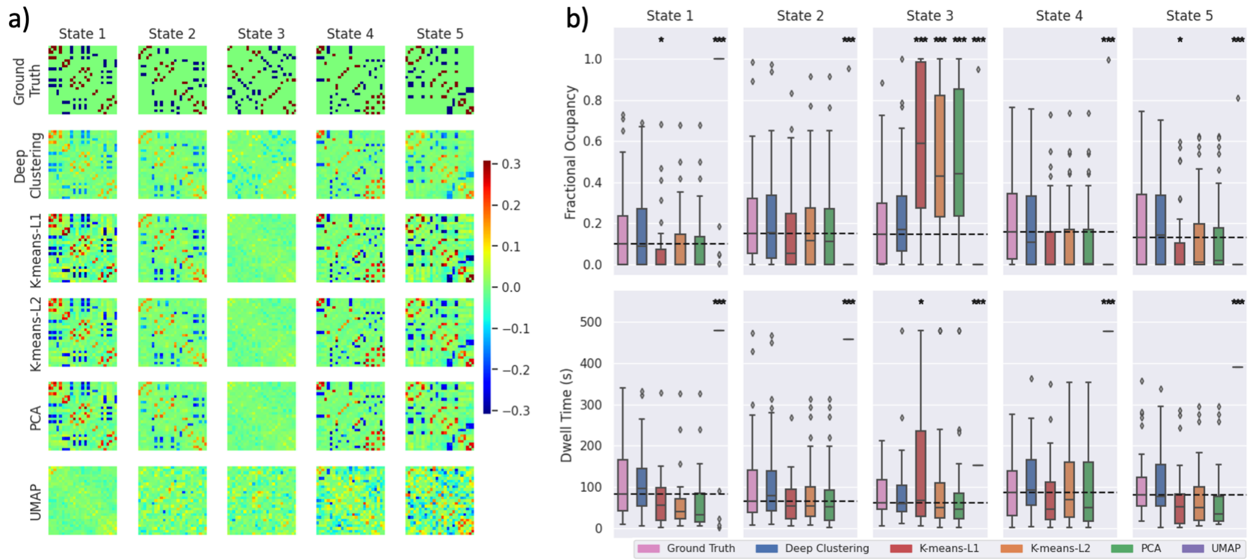

Supplementary Figure 11: Clustering results from one run of each dimensionality reduction method applied to synthetic data processed with a 60 s window. a) State FC matrices are plotted with connectivity indicated by the colour bar. b) The distribution across subjects of fractional occupancy and dwell time measurements are plotted for each state. Boxes show the interquartile range, with a line for the median. The median of the ground truth is shown as a dashed line across each plot for comparison with other methods. Whiskers extend to the range of the data, not including outliers which are shown as diamonds. Significant differences from the true distributions, measured by unpaired two-tailed t-tests, are indicated as follows: $*p < 0.01$, $**p < 0.001$, $***p < 0.0001$.

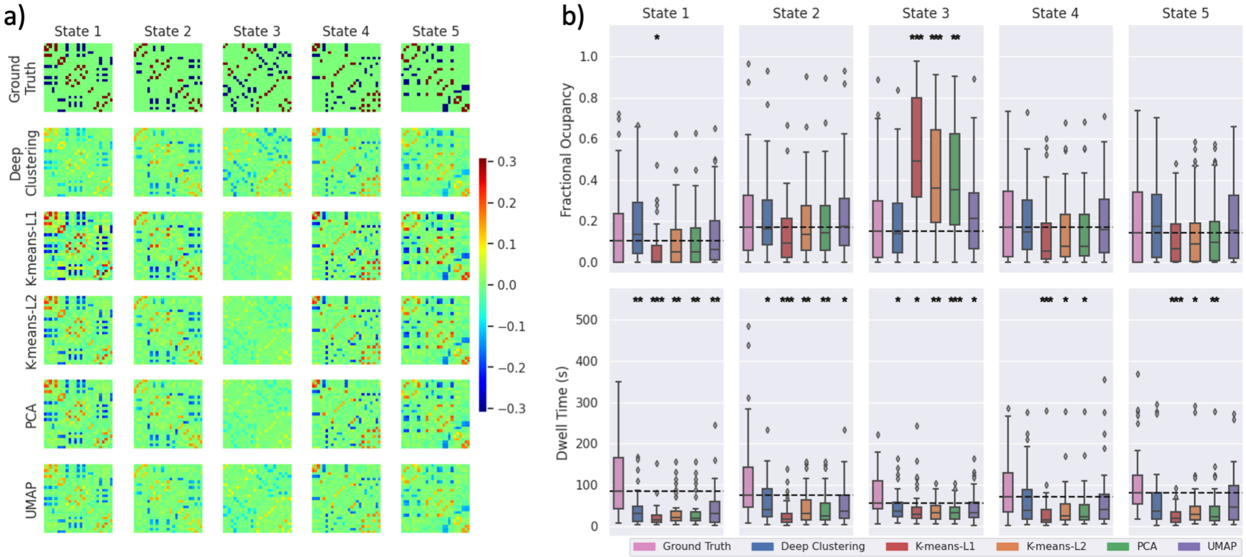

Supplementary Figure 12: Clustering results from one run of each dimensionality reduction method applied to synthetic data processed with a Hamming window. a) State FC matrices are plotted with connectivity indicated by the colour bar. b) The distribution across subjects of fractional occupancy and dwell time measurements are plotted for each state. Boxes show the interquartile range, with a line for the median. The median of the ground truth is shown as a dashed line across each plot for comparison with other methods. Whiskers extend to the range of the data, not including outliers which are shown as diamonds. Significant differences from the true distributions, measured by unpaired two-tailed t-tests, are indicated as follows: $*p < 0.01$, $**p < 0.001$, $***p < 0.0001$.

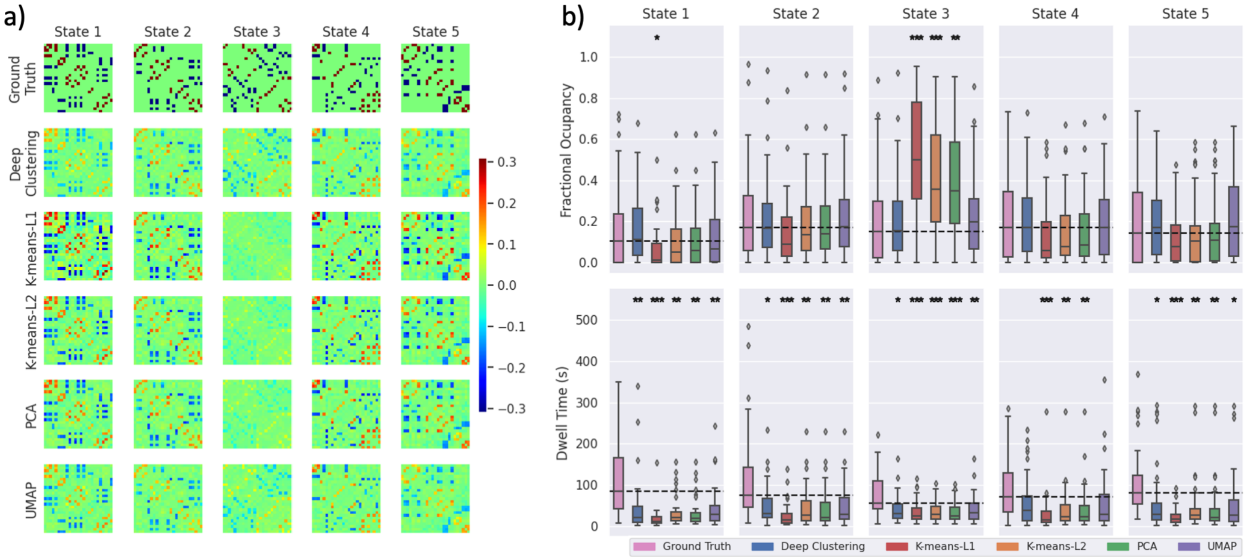

Supplementary Figure 13: Clustering results from one run of each dimensionality reduction method applied to synthetic data processed with a Hanning window. a) State FC matrices are plotted with connectivity indicated by the colour bar. b) The distribution across subjects of fractional occupancy and dwell time measurements are plotted for each state. Boxes show the interquartile range, with a line for the median. The median of the ground truth is shown as a dashed line across each plot for comparison with other methods. Whiskers extend to the range of the data, not including outliers which are shown as diamonds. Significant differences from the true distributions, measured by unpaired two-tailed t-tests, are indicated as follows: $*p < 0.01$, $**p < 0.001$, $***p < 0.0001$.

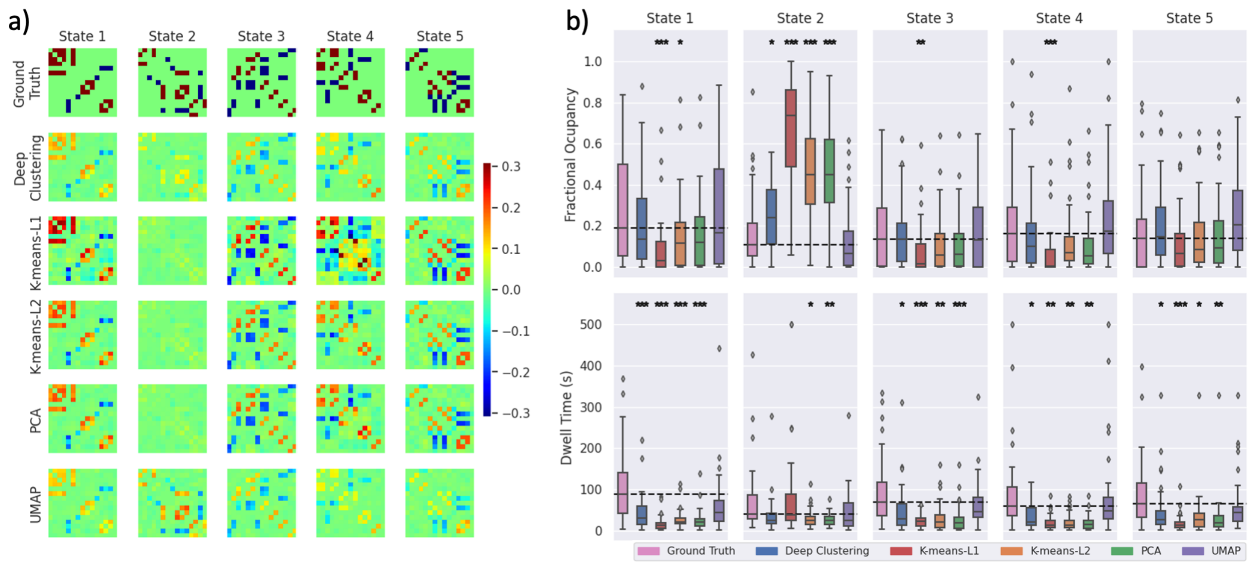

Supplementary Figure 14: Clustering results from one run of each dimensionality reduction method applied to synthetic data with 15 nodes. a) State FC matrices are plotted with connectivity indicated by the colour bar. b) The distribution across subjects of fractional occupancy and dwell time measurements are plotted for each state. Boxes show the interquartile range, with a line for the median. The median of the ground truth is shown as a dashed line across each plot for comparison with other methods. Whiskers extend to the range of the data, not including outliers which are shown as diamonds. Significant differences from the true distributions, measured by unpaired two-tailed t-tests, are indicated as follows: $*p < 0.01$, $**p < 0.001$, $***p < 0.0001$.

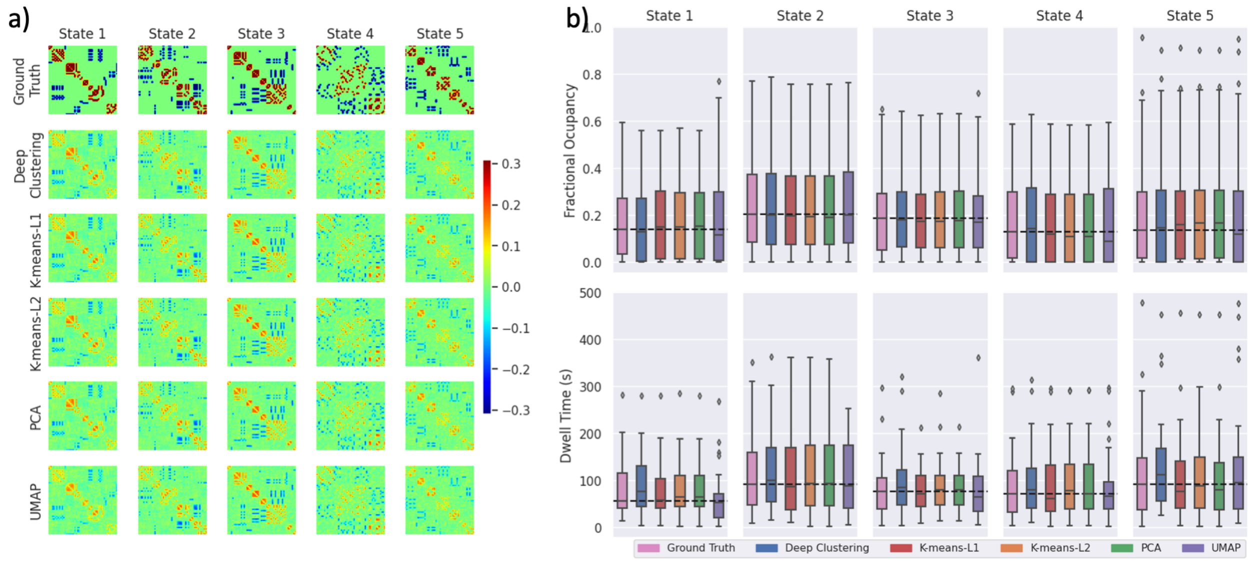

Supplementary Figure 15: Clustering results from one run of each dimensionality reduction method applied to synthetic data with 50 nodes. a) State FC matrices are plotted with connectivity indicated by the colour bar. b) The distribution across subjects of fractional occupancy and dwell time measurements are plotted for each state. Boxes show the interquartile range, with a line for the median. The median of the ground truth is shown as a dashed line across each plot for comparison with other methods. Whiskers extend to the range of the data, not including outliers which are shown as diamonds. Significant differences from the true distributions, measured by unpaired two-tailed t-tests, are indicated as follows: $*p < 0.01$, $**p < 0.001$, $***p < 0.0001$.

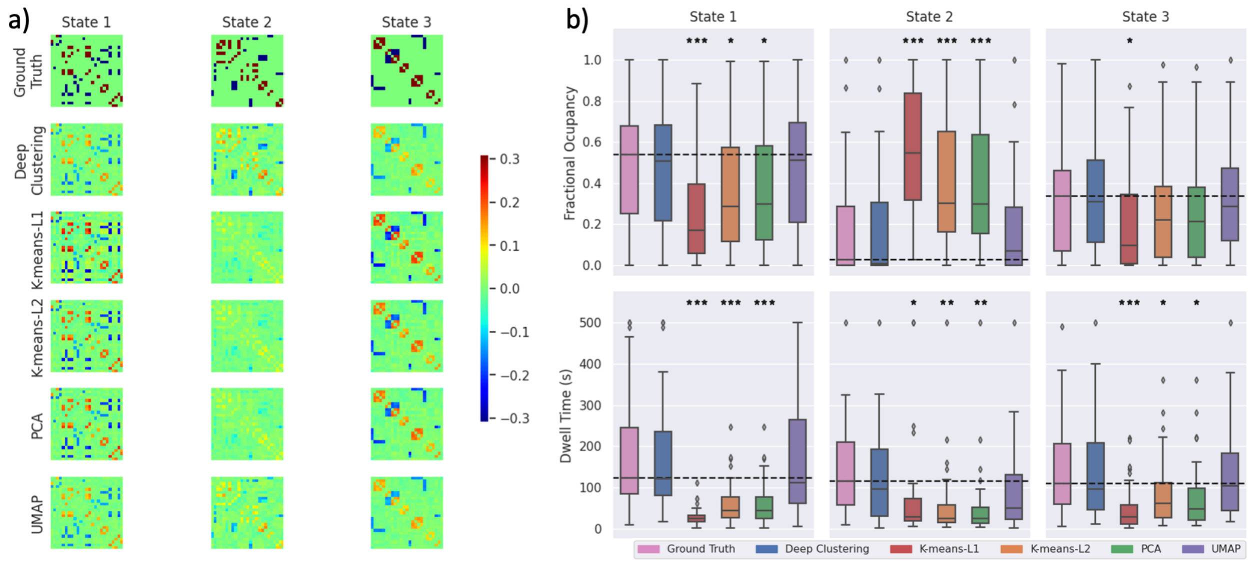

Supplementary Figure 16: Clustering results from one run of each dimensionality reduction method applied to synthetic data with 3 states. a) State FC matrices are plotted with connectivity indicated by the colour bar. b) The distribution across subjects of fractional occupancy and dwell time measurements are plotted for each state. Boxes show the interquartile range, with a line for the median. The median of the ground truth is shown as a dashed line across each plot for comparison with other methods. Whiskers extend to the range of the data, not including outliers which are shown as diamonds. Significant differences from the true distributions, measured by unpaired two-tailed t-tests, are indicated as follows: $*p < 0.01$, $**p < 0.001$, $***p < 0.0001$.

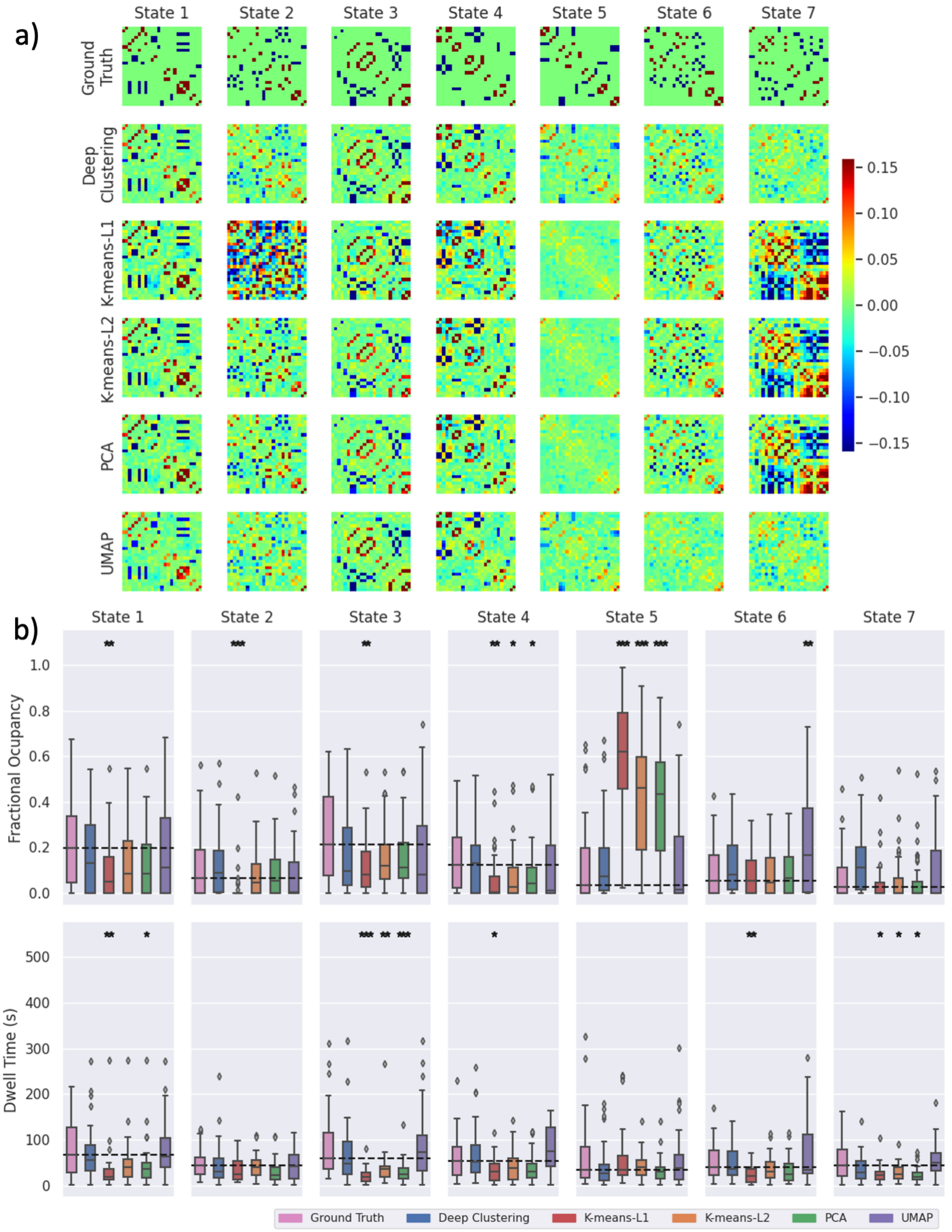

Supplementary Figure 17: Clustering results from one run of each dimensionality reduction method applied to synthetic data with 7 states. a) State FC matrices are plotted with connectivity indicated by the colour bar. b) The distribution across subjects of fractional occupancy and dwell time measurements are plotted for each state. Boxes show the interquartile range, with a line for the median. The median of the ground truth is shown as a dashed line across each plot for comparison with other methods. Whiskers extend to the range of the data, not including outliers which are shown as diamonds. Significant differences from the true distributions, measured by unpaired two-tailed t-tests, are indicated as follows: $*p < 0.01$, $**p < 0.001$, $***p < 0.0001$.

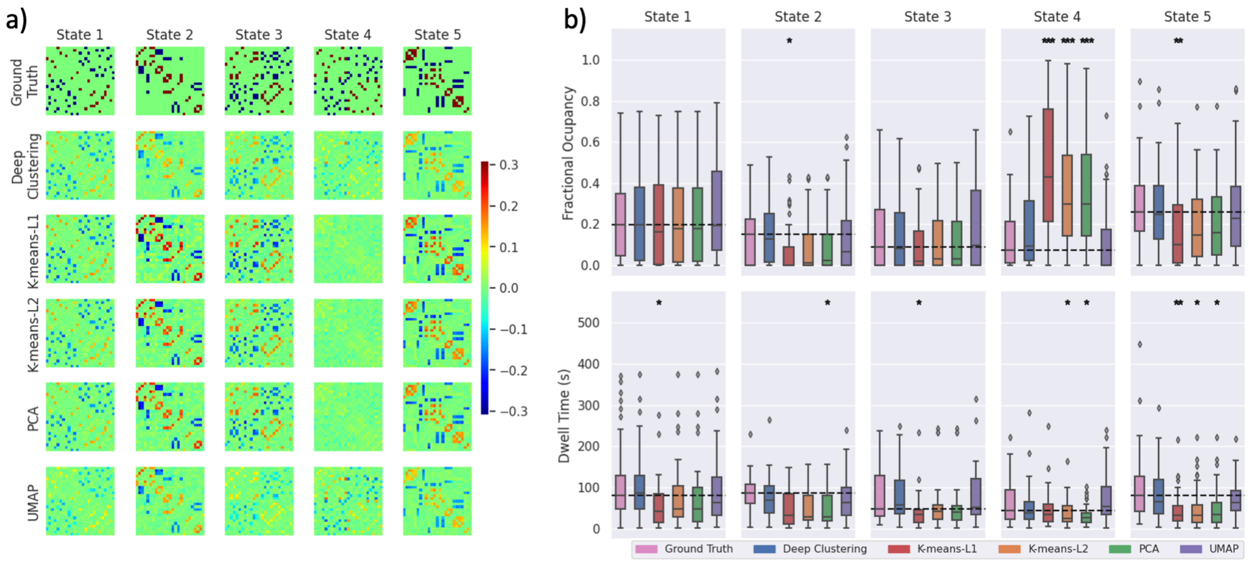

Supplementary Figure 18: Clustering results from one run of each dimensionality reduction method applied to synthetic data constructed with the Windkessel-Balloon HRF. a) State FC matrices are plotted with connectivity indicated by the colour bar. b) The distribution across subjects of fractional occupancy and dwell time measurements are plotted for each state. Boxes show the interquartile range, with a line for the median. The median of the ground truth is shown as a dashed line across each plot for comparison with other methods. Whiskers extend to the range of the data, not including outliers which are shown as diamonds. Significant differences from the true distributions, measured by unpaired two-tailed t-tests, are indicated as follows: $*p < 0.01$, $**p < 0.001$, $***p < 0.0001$.

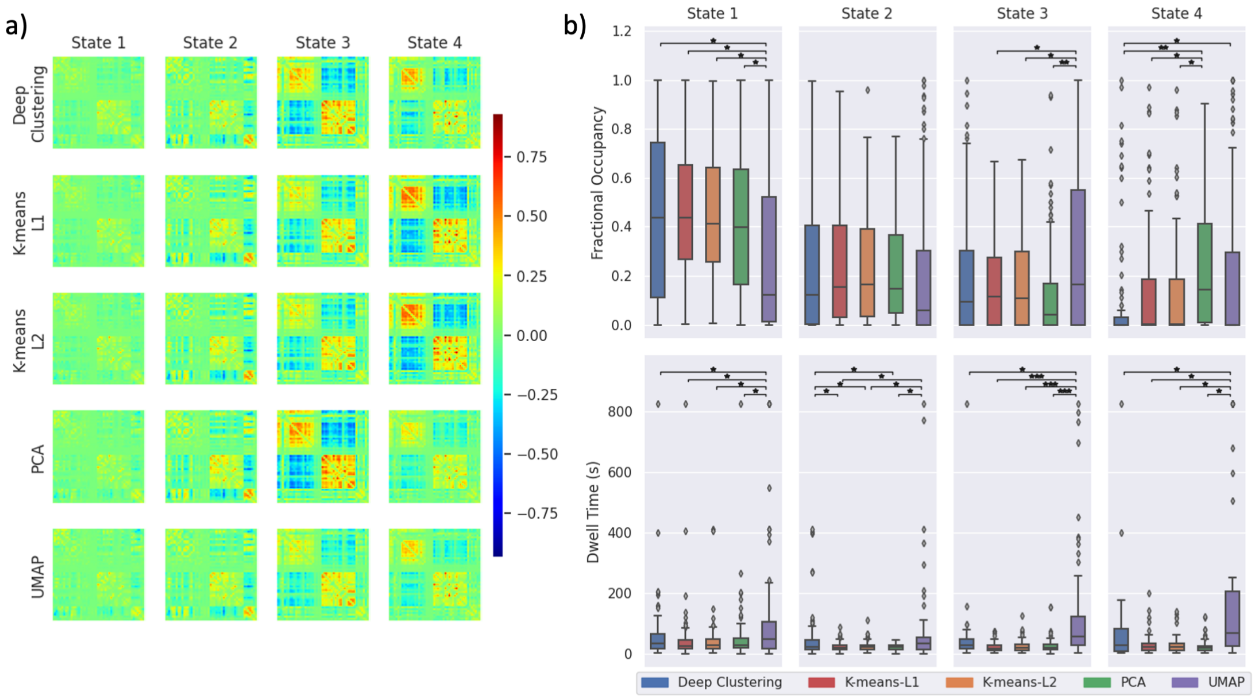

Supplementary Figure 19: Clustering results from each dimensionality reduction method applied to the second group of 100 subjects from the Human Connectome Project. a) State FC matrices resulting from each method, with connectivity indicated by the colour bar. b) Fractional occupancy and dwell time measurements across subjects are shown for each state. Boxes show the interquartile range with a line for the median. Whiskers extend to the range of the data, not including outliers which are shown as diamonds. Measurements were compared using a one-way ANOVA, with post hoc pairwise comparison using two-tailed t-tests. FDR-corrected: $*p < 0.05$, $**p < 0.001$, $***p < 0.0001$.

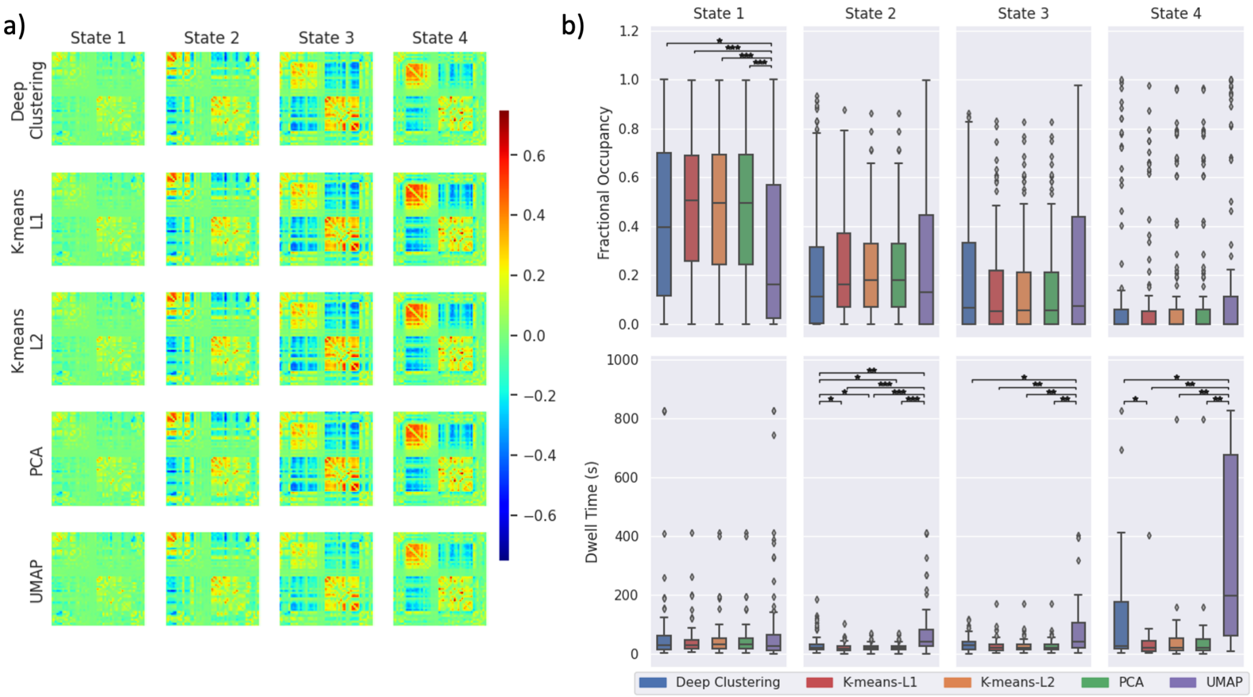

Supplementary Figure 20: Clustering results from each dimensionality reduction method applied to the third group of 100 subjects from the Human Connectome Project. a) State FC matrices resulting from each method, with connectivity indicated by the colour bar. b) Fractional occupancy and dwell time measurements across subjects are shown for each state. Boxes show the interquartile range with a line for the median. Whiskers extend to the range of the data, not including outliers which are shown as diamonds. Measurements were compared using a one-way ANOVA, with post hoc pairwise comparison using two-tailed t-tests. FDR-corrected: $*p < 0.05$, $**p < 0.001$, $***p < 0.0001$.

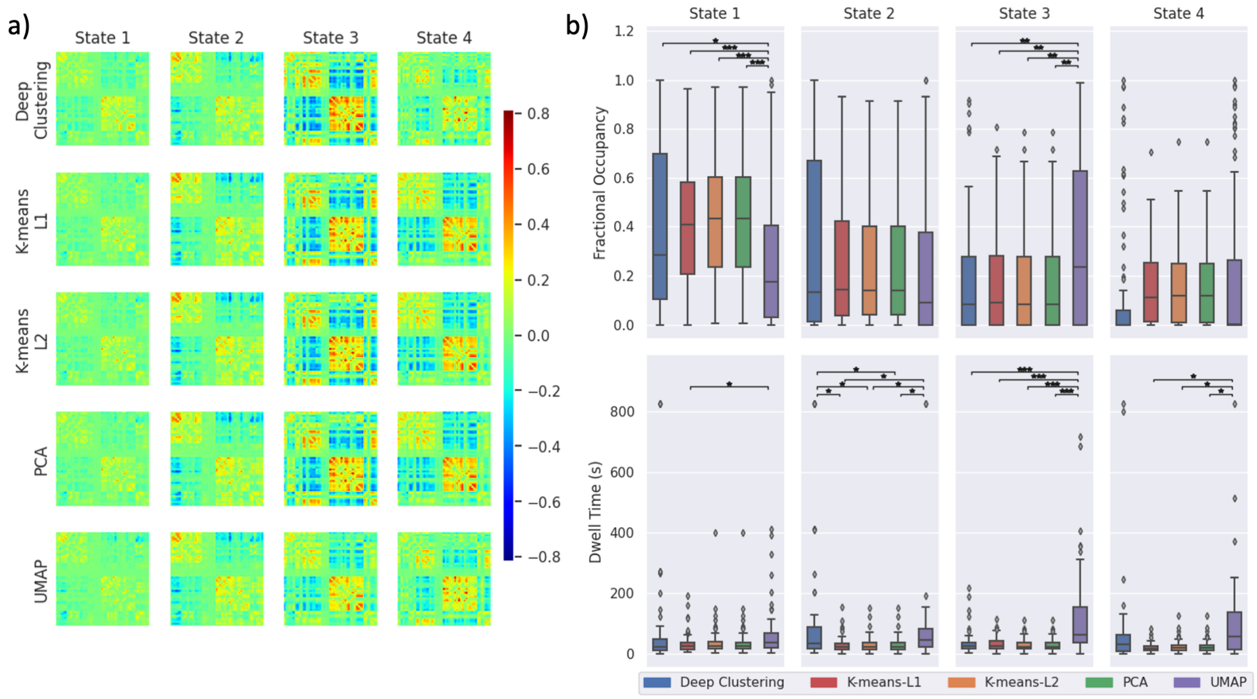

Supplementary Figure 21: Clustering results from each dimensionality reduction method applied to the fourth group of 100 subjects from the Human Connectome Project. a) State FC matrices resulting from each method, with connectivity indicated by the colour bar. b) Fractional occupancy and dwell time measurements across subjects are shown for each state. Boxes show the interquartile range with a line for the median. Whiskers extend to the range of the data, not including outliers which are shown as diamonds. Measurements were compared using a one-way ANOVA, with post hoc pairwise comparison using two-tailed t-tests. FDR-corrected: $*p < 0.05$, $**p < 0.001$, $***p < 0.0001$.

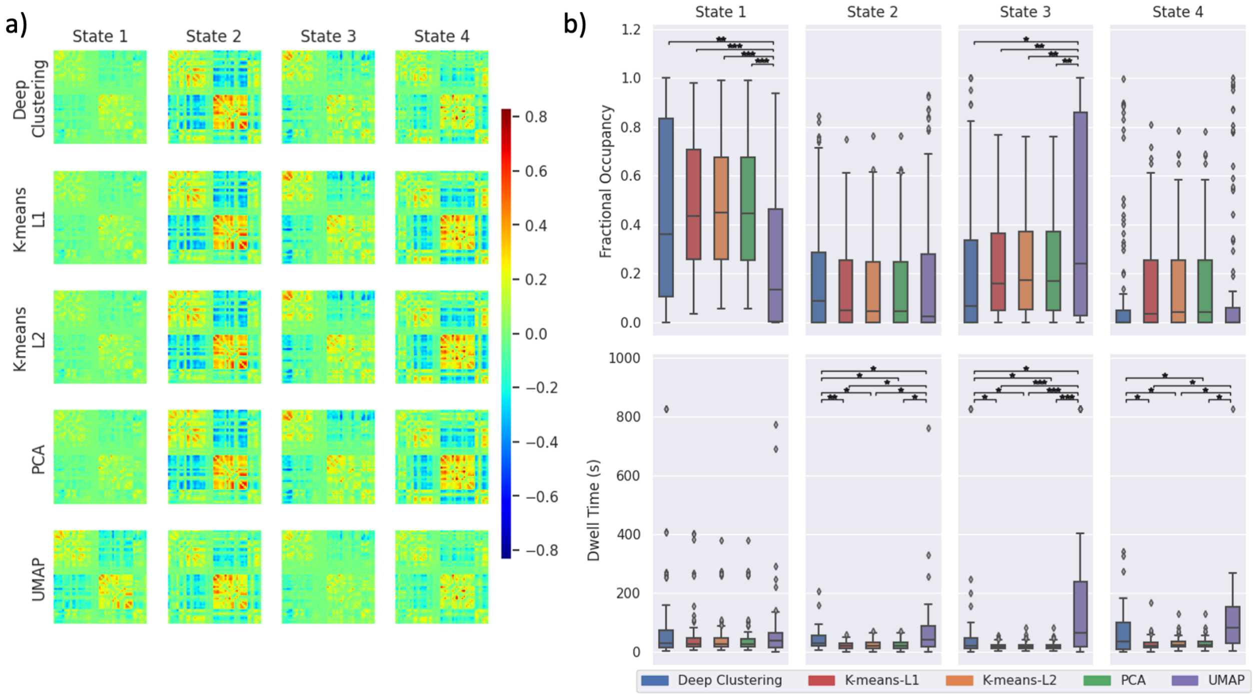

Supplementary Figure 22: Clustering results from each dimensionality reduction method applied to the fifth group of 100 subjects from the Human Connectome Project. a) State FC matrices resulting from each method, with connectivity indicated by the colour bar. b) Fractional occupancy and dwell time measurements across subjects are shown for each state. Boxes show the interquartile range with a line for the median. Whiskers extend to the range of the data, not including outliers which are shown as diamonds. Measurements were compared using a one-way ANOVA, with post hoc pairwise comparison using two-tailed t-tests. FDR-corrected: $*p < 0.05$, $**p < 0.001$, $***p < 0.0001$.

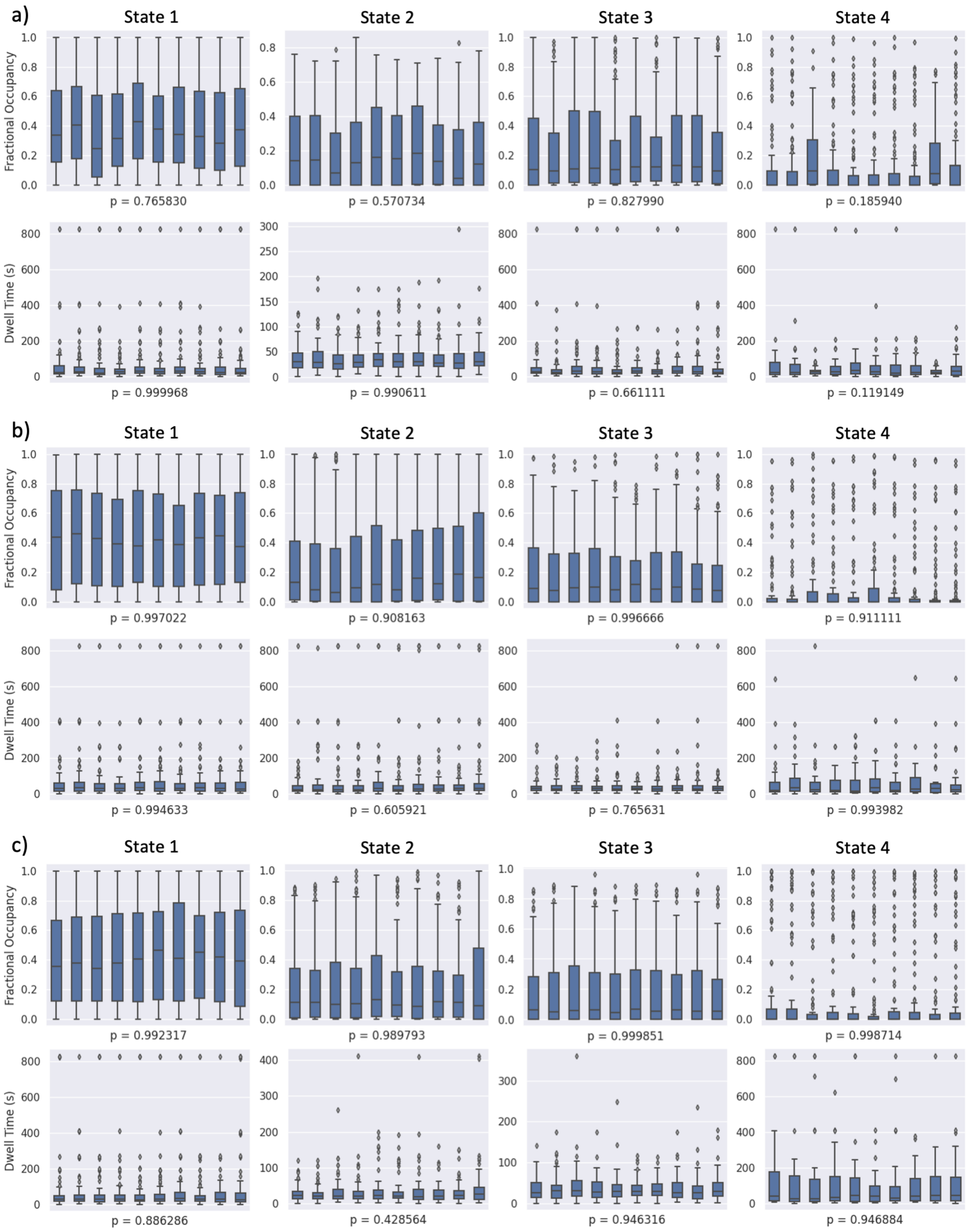

Supplementary Figure 23 (continues on next page)

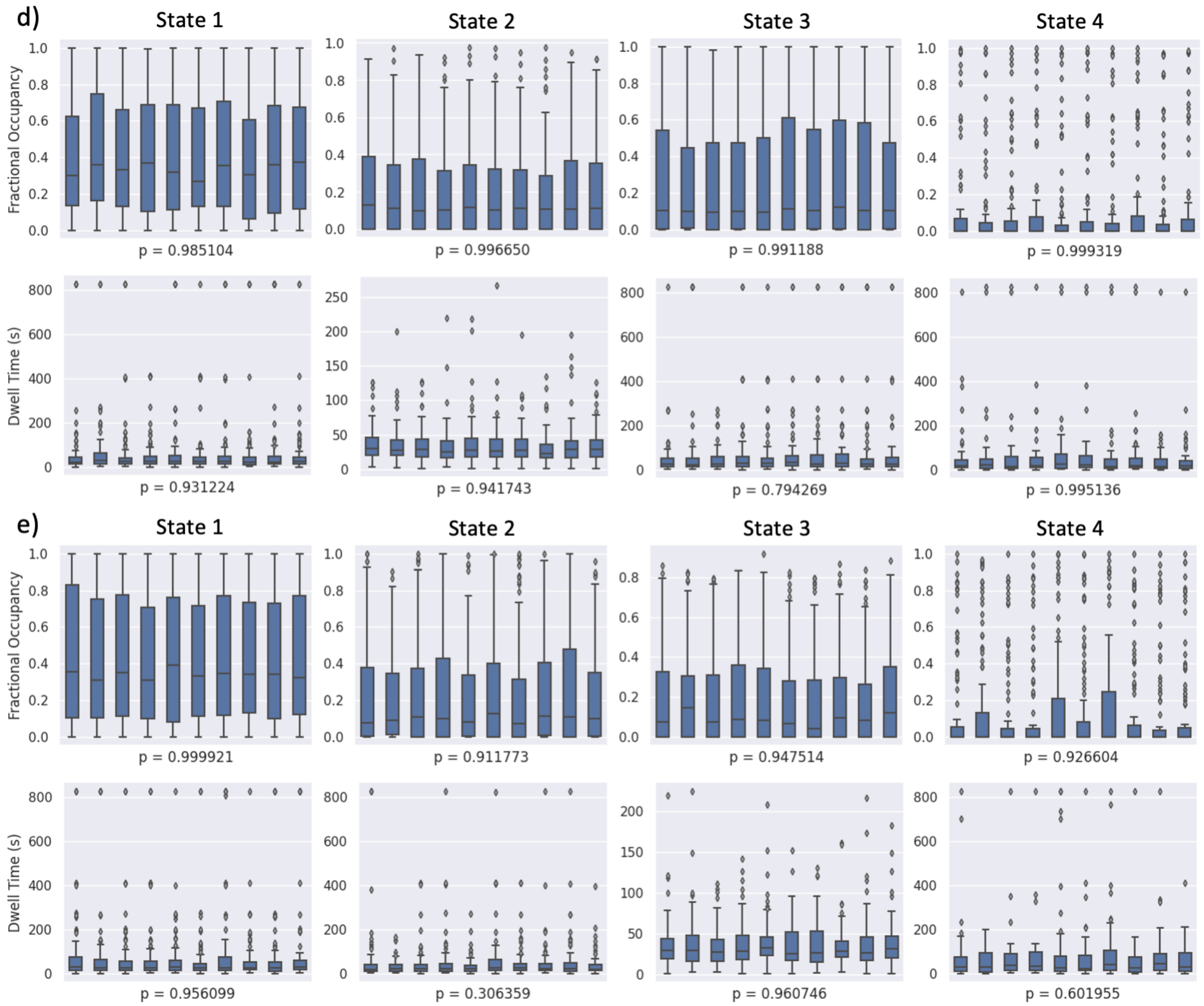

Supplementary Figure 23 (cont.): Repeated runs of deep clustering applied to real data. Each subfigure (a–e) shows results from a different group of 100 participants from the Human Connectome Project. Distributions of fractional occupancy and dwell time are shown for each run of deep clustering (one box per run), with boxes showing the interquartile range with a line for the median and whiskers extending to the range of the data, not including outliers which are shown as diamonds. Each plot shows the p-value of a one-way ANOVA comparing the distribution of measurements across repeated runs. None are significant, indicating that deep clustering gives reproducible measurements of fractional occupancy and dwell time across repeated runs.
